# Candidate Gene Analysis of Tooth Agenesis Identifies Novel Mutations in Six Genes and Suggests Significant Role for WNT and EDA Signaling and Allele Combinations

**DOI:** 10.1371/journal.pone.0073705

**Published:** 2013-08-22

**Authors:** Sirpa Arte, Satu Parmanen, Sinikka Pirinen, Satu Alaluusua, Pekka Nieminen

**Affiliations:** 1 Institute of Dentistry, University of Helsinki, Helsinki, Finland; 2 Department of Oral and Maxillofacial Diseases, Helsinki University Central Hospital, Helsinki, Finland; New York Medical College, United States of America

## Abstract

Failure to develop complete dentition, tooth agenesis, is a common developmental anomaly manifested most often as isolated but also as associated with many developmental syndromes. It typically affects third molars or one or few other permanent teeth but severe agenesis is also relatively prevalent. Here we report mutational analyses of seven candidate genes in a cohort of 127 probands with non-syndromic tooth agenesis. 82 lacked more than five permanent teeth excluding third molars, called as oligodontia. We identified 28 mutations, 17 of which were novel. Together with our previous reports, we have identified two mutations in *MSX1*, *AXIN2* and *EDARADD*, five in *PAX9*, four in *EDA* and *EDAR*, and nine in *WNT10A*. They were observed in 58 probands (44%), with a mean number of missing teeth of 11.7 (range 4 to 34). Almost all of these probands had severe agenesis. Only few of the probands but several relatives with heterozygous genotypes of *WNT10A* or *EDAR* conformed to the common type of non-syndromic tooth agenesis, incisor-premolar hypodontia. Mutations in *MSX1* and *PAX9* affected predominantly posterior teeth, whereas both deciduous and permanent incisors were especially sensitive to mutations in *EDA* and *EDAR*. Many mutations in *EDAR*, *EDARADD* and *WNT10A* were present in several families. Biallelic or heterozygous genotypes of *WNT10A* were observed in 32 and hemizygous or heterozygous genotypes of *EDA*, *EDAR* or *EDARADD* in 22 probands. An *EDARADD* variant were in seven probands present together with variants in *EDAR* or *WNT10A*, suggesting combined phenotypic effects of alleles in distinct genes.

## Introduction

Human dentition consists of different types of deciduous and permanent teeth with unique hard tissues, specific shapes and reproducible timetable of development and eruption. The development is regulated by intricate cell and tissue interactions and genetic networks, the components of which include the same signaling pathways active in all development and that of ectodermal organs in special [1]. The complexity of development renders the dentition vulnerable by genetic and environmental factors which may affect the number, eruption or structure of teeth [2]. Failure to develop all teeth, tooth agenesis (also called hypodontia or oligodontia) is the most common dental anomaly. One fourth of us fail to develop all third molars (wisdom teeth), more than 5% lacks some of the other permanent teeth (hypodontia), and also more severe agenesis is relatively common [3,4]. Agenesis of six or more permanent teeth apart from third molars (also called oligodontia) happens in 1 in 1000 [5–7]. Despite tooth agenesis may express itself in different forms and especially the severe forms may affect any teeth, the most commonly missing teeth are the ones that develop latest in each tooth class [4], suggesting that typically tooth agenesis is explained by quantitative defects affecting the whole tooth class [8].

Several lines of observation have indicated that tooth agenesis is predominantly genetically determined. It is often familial, and it is present in inherited malformation syndromes [8,9]. During the era of molecular genetics, the underlying gene mutations have been identified in numerous syndromic forms and in severe isolated forms which follow various modes of inheritance [8]. The identified mutations affect all major signaling pathways and genetic networks mediating their effects, showing that tooth agenesis may arise by defects in distinct aspects of tooth development. Mutations in different genes typically cause phenotypes characteristic in terms of severity and affected teeth. First mutations in isolated tooth agenesis were detected in *MSX1* (ENSG00000163132, OMIM 106600) and *PAX9* (ENSG00000198807, OMIM 604625) and subsequently numerous heterozygous loss of function mutations have been reported in these genes coding for transcription factors active in the dental mesenchyme [10–12]. However, these mutations are rare, and usually limited to single families. These dominant mutations were complemented by identification of similar mutations in *AXIN2* (ENSG00000168646, OMIM 608615) [13]. More recently, mutations in isolated tooth agenesis have been identified in *EDA* (ENSG00000158813, OMIM 313500) and *WNT10A* (ENSG00000135925, OMIM 606268) [14–19]. *EDA* codes for a TNF-like signal molecule ectodysplasin active in the epithelium and was identified as the mutated gene in X-linked form of anhidrotic ectodermal dysplasia (OMIM 305100) [20]. First mutations in *WNT10A* were identified in patients with another ectodermal dysplasia, recessive odonto-onycho-dermal dysplasia (OODD, OMIM 257980), and an allelic disease Schöpf-Schulz-Passarge syndrome (SSPS, OMIM 224750), and most recently homozygous and heterozygous mutations have been identified in patients with isolated tooth agenesis [17–19,21,22]. The importance of *WNT10A* for tooth agenesis is in line with its rather specific expression in the epithelial signaling centers important for tooth development [23,24]. Despite this progress, the underlying genetic factors for a significant part of severe tooth agenesis and especially the more common forms, including incisor premolar hypodontia [25], remains unexplained.

We have previously described phenotypes and inheritance as well as causative mutations in different types of tooth agenesis [13,25–27]. In this report, we describe results from a mutational analysis of the most important candidate genes *MSX1*, *PAX9*, *AXIN2*, *EDA*, *EDAR*, *EDARADD*, and *WNT10A* in our cohort of Finnish patients.

## Patients and Methods

### Recruitment and phenotypic analysis

The probands were research patients at the Institute of Dentistry, University of Helsinki, and patients referred to the Helsinki University Central Hospital (HUCH) because of need of dental treatment. They were informed of the purpose and procedures of the research with discussion and with information leaflets and they, or in case of children under 15 years of age, their parents, signed a form of an informed consent. The relatives were also asked to participate in the study. All clinical and molecular genetic studies were conducted according to the principles expressed in the Declaration of Helsinki and under permission from the Ethics Committee, Department of Surgery, HUCH. Diagnosis of tooth agenesis was confirmed by clinical and radiographic examination or from previous documents. The general health, including ectodermal features and other syndromic indications as well as history of cancer, were assessed by clinical examination and interviews. The patients with diagnosis or suspected syndrome were excluded from this study. The genomic DNA was isolated from blood samples in the DNA isolation core unit of the National Institute for Health and Welfare, Biomedicum Helsinki, or from buccal swabs in the laboratory of the research group by using Qiaamp DNA mini kit (Qiagen).

### Mutational analysis

For a mutational analysis by sequencing of exons of *MSX1*, *PAX9*, *AXIN2*, *EDA*, *EDAR* (ENSG00000135960), *EDARADD* (ENSG00000186197), *WNT6* (ENSG00000155196), *WNT10A* and *WNT10B* (ENSG00000169884) primers were planned to span exons and at least 30 bp of the flanking intronic sequences with Primer 3 software (http://frodo.wi.mit.edu/). The target was amplified by PCR and the products purified with ExoSAP-method (USB) and used as templates in the sequencing reaction by ABI BDRR reagent v 3.1 (Applied Biosystems). The sequencing products were subjected to capillary electrophoresis in the Biomedicum Helsinki Molecular medicine sequencing laboratory. The results were compared with known genomic sequences for each gene (BLAST, http://www.ncbi.nlm.nih.gov/blast; *MSX1*: NT_006051.15; *PAX9*: NT_026437.10; *AXIN2*: NT_010783.15; *EDA*: NT_011669.16; *EDAR*: NT_022171.14; *EDARADD*: NT_004836.17; *WNT10A* and *WNT6*: NT_005403.17; *WNT10B*: NT_029419.12) and the observed deviation from reference stored in a proprietary database. For the primer sequences and PCR and sequencing conditions, see Table 1. Observed missense variants were subjected to bioinformatic and conservation analysis by SIFT (http://sift.jcvi,org) and PolyPhen2 (http://genetics.bwh.harvard.edu/pph2/index.shtml). Eventual effect on splicing sites were studied by NetGene2 (http://www.cbs.dtu.dk/services/NetGene2/) and Berkeley splicing site prediction tool (http://www.fruitfly.org/seq_tools/splice.html) and effects on functional sites by ELM (Eukaryotic linear motif resource (http://elm.eu.org/).

## Results

Altogether 127 probands were studied in this study. 54 of the probands were males, and 73 were females. Including the four families with severe phenotypes that we have reported earlier [13,26,27], our whole cohort thus included 131 probands. The incisor premolar hypodontia as well as incisor hypodontia families that we have described earlier were included in the cohort [25,28]. The great majority of probands had a severe phenotype (Table 1). 82 of the current study cohort fulfilled the definition of oligodontia and the mean number of missing permanent teeth excluding the third molars in this group was 9.0 (range 6 to 20). Significant proportion of the other 45 probands had phenotypes that were more severe than the common types of incisor premolar hypodontia: the mean number of missing permanent teeth excluding the third molars was 3.2 and 18 probands lacked five or four such teeth. Of the whole cohort, agenesis in the deciduous dentition could be verified in 21 probands, and most of these were incisors. In some probands, minor ectodermal features like thin hair, atopic skin or soft nails were recorded. Hypohidrosis or hyperhidrosis or abnormalities of the tongue papillae were not observed. In most cases in which phenotypes of the relatives were available, some extent of tooth agenesis was observed in the parents, siblings or other relatives. In several of these families autosomal dominant inheritance was suggested and many were consistent with recessive inheritance but in many families the mode of inheritance was not obvious. Results from the mutational analysis were in several families not consistent with the original assumption on the mode of inheritance.

**Table 1:** Summary of probands with tooth agenesis.

n permanent tooth agenesis*)	probands**)	involvement of 3rd molars	deciduous teeth
1	1	0	0
2	16	4	2
3	10	2	2
4	10	4	4
5	8	5	1
6	17	9	4
7	15	10	1
8	18	15	1
9	5	3	1
10	5	2	1
11	7	6	0
12	6	3	0
13	4	3	1
14	2	2	0
15	1	0	0
> 15	6	5	3
all	131	73	21
>= 6	86	58	12
<= 5	45	15	9
7.2	mean (all)		
6	median (all)		

* excluding third molars

** including the four probands reported earlier (see references in the text)

During this study, the mutational analysis revealed suggested causative mutations in 54 probands and 46 of their relatives. Most of the mutations were novel. The mutations and their bioinformatic analyses are described in Table 2 and the associated genotypes and phenotypes in Table 3 and Table 2. Except for one, c.308C>T (p. S103F) in *EDARADD*, none of the mutations were present in more than 100 Finnish unrelated healthy control persons.

**Table 2:** Identified mutations and their bioinformatic analysis.

ORF	protein	ex	dbSNP	probands		relatives		cont	domain	bioi.
				biall	het	biall	het	het		
*MSX1*										
c. 665-666insA	N222KfsX118	2	novel		1			0	C-terminus	na
c.708delG	K237SfsX2	2	novel		1		1	0	C-terminus	na
*PAX9*										
c.140G>C	R47P	2	novel		1			0	paired	SP**)
c. 167T>C	I56T	2	novel		1			0	paired	SP
c.340A>T	K114X	2	rs104894467		1			0	paired	na
c.406C>T	Q136X	2	novel		1			0	paired	na
*EDA*										
c.612-29del18bp	IPGIPG	7	novel	1*)				0	collagen-like	na
	205-210del									
c.901-904delTACT	T301SfsX5	10	novel		1			0	TNF-hom.	na
c.1069C>T	R357W	11	novel	1*)				0	TNF-hom.	SP
c.1133C>T	T378M	11	novel		1			0	TNF-hom.	SP
*EDAR*										
c.973C>T	R325W	11	novel		3		4	0	death-like	SP
c.1073G>A	R358Q	12	novel		1		1	0	death-like	SP
c.1135G>A	E379K	12	novel		3		2	0	death	SP
c.1172T>A	M391K	12	novel		1		1	0	death	SP
*EDARADD*										
c.308C>T	S103F	7	rs114632254		11***)			4		Sp
c.508C>T	R170W	7	novel		1			0	death	SP
*WNT10A*										
c.208C>T	R70W	2	rs146460077		1		1	0	NTD, is3****)	SP
c.337C>T	R113C	2	rs141074983	4	2		2	0	NTD	Sp
c.433G>A	V145M	3			1		6	0	NTD	SP
c.433G>T	V145L	3	novel		1			0	NTD	SP
c.460C>A	L154M	3	rs148187600		1		3	0	NTD	SP
c.493G>A	G165R	3	rs77583146		6		2	0	NTD	RRGD
										>RRRD
c.579-592del	E194AfsX28	3	novel	1			1	0	NTD	na
GGAACACCCAGCCC										
c.664G>T	E222X	3	novel	1		1	1	0	NTD	na
c.682T>A	F228I	3	rs121908120	9	9	1	16	0	NTD, is3	SP

Variant numbers are based on the following cDNA entries in Genbank: *MSX1*, NM_002448.3; *PAX9*, NM_006194.3; *EDA*, NM_001399.4; *EDAR*, NM_022336.3; *EDARADD*, NM_145861.2; *WNT10A*, NM_025216.2.

ex, exon; biall, biallelic, i.e. homozygous or compound heterozygous;

het, heterozygous; cont, controls; bioi, bioinformatics

*)hemizygous

**) S, affects protein function (SIFT); P, probably damaging (PolyPhen2); p, possibly damaging (PolyPhen2); na, not applicable

***) Seven of these were obserbed in combination with a mutation in *EDAR* or *WNT10A*

****)NTD, N-terminal a-helical bundle; is3, putative interaction site 3

**Table 3:** Candidate gene genotypes and tooth agenesis phenotypes.

fam	sex	age	genotype (protein)	inherit- ance	n ta all	n ta per	jaw	right														left	
								8	7	6	5	4	3	2	1	1	2	3	4	5	6	7	8
250	f	10	MSX1:	ad (de	14	11	U	*			*	*					*		*	*			
			N222KfsX118/-	novo)			L	*	*		*				*	*				*		*	*
219	f	15	MSX1:K237SfsX2/-	ad	12	8	U	*			*	*		**p**			**p**		*	*			*
							L	*			*				*	*				*			*
283	f	11	PAX9:R47P/-	ad	11	7	U	*	*								**p**			*		*	*
							L	*			*				*	*				*			*
272	f	23	PAX9:I56T/-	ad	9	5	U	*	*	*											*	*	*
							L	*														*	*
204	f	11	PAX9:K114X/-	ad (de	9	5	U	*	*	*											*	*	*
				novo)			L	*														*	*
278	f	28	PAX9:Q136X/-	ad	11	7	U	*	*	*	*										*	*	*
							L	*	*													*	*
41	m	12	EDA:	Xl (de	5	3	U							*			*						
			IPGIPG	novo)			u							*			*						
			205-210del				l																
							L							**c**	**c**	*	**c**						
412	f	6	EDA:T301SfsX5/-	Xl	29	18	U	**?**			*	*	*	*			*	*	*	*			**?**
							u					*		*			*		*	*			
							l					*	**c**	**p**	*	*	*	**c**	*	*			
							L				*	*	*	*	*	*	*	*	*	*			
46	m	13	EDA:R357W	Xl	18	8	U	*				*		*		**c**	*		*				*
							u					*		*			*		*				
							l							*	*	*	*						
							L							*	*	*	*						
406	f	17	EDA:T378M/-	Xl	8	8	U							*			*		*				
							L							*	*	*	*		*				
47	f	6	EDAR:R325W/-	ad?	4	2	U	**?**						*			*						**?**
							u							*			*						
							l																
							L	**?**															**?**
52	m	13	EDAR:R325W/-	ac	5	4	U										*						
			; EDARADD:S103F/-				u																
							l							*									
							L							*	*	*							
266	f	19	EDAR:R325W/-	ac	15	9	U	*			*		*	*			*	*		*			*
			; WNT10A:G165R/-				u							*			*						
			; EDARADD:S103F/-				l																
							L	*						*			*			*			*
67	m	5	EDAR:R358Q/-	ad?	10	5	U	**?**						*			*						**?**
							u							*			*						
							l							*		*	*						
							L	**?**							*	*	*						**?**
49	f	17	EDAR:E379K/-	ad?	8	4	U							*			*						
							u							*			*						
							l							*			*						
							L							*			*						
108	m	7	EDAR:E379K/-	ad	4	3	U	**?**						*			*						**?**
							u										*						
							l																
							L	**?**			*												**?**
221	f	10	EDAR:E379K/-	ad?	10	10	U				*	*	*	*			*	*		*			
							L					*							*	*			
402	f	18	EDAR:M391K/-	ad	13	6	U							*			*						
							u							*			*						
							l								*	*	*						
							L	*			*			*			*			*			*
19	m	29	EDARADD:S103F/-	?	6	2	U	*						**p**			**p**						*
							L	*	*													*	*
230	f	35	EDARADD:S103F/-	?	10	6	U	*			*									*			*
							L	*			*								*	*		*	*
245	f	21	EDARADD:S103F/-	?	14	12	U				*	*	*	*			*	*	*	*			
							L	*			*	*							*	*			*
269	f	21	EDARADD:S103F/-	?	12	8	U	*			*			*			*			*			*
							L	*	*		*									*		*	*
209	f	24	EDARADD:R170W/-	ad?	11	7	U	*				*		**p**			**p**		*	*			*
							L	*			*	*							*	*			*
276	m	43	WNT10A:R70W/-	ac	10	6	U	*			*			*						*			*
			; EDARADD:S103F/-				L	*			*									*		*	*
253	m	54	WNT10A:	ar	12	8	U	*			*	*							*	*			*
			R113C/R113C				L	*			*	*							*	*			*
68	m	22	WNT10A:	ar	14	12	U				*		*	*			*	*		*			
			R113C/F228I				L	*					*	*	*	*	*	*					*
265	f	8	WNT10A:	ar	9	5	U	*						**?**			*						*
			R113C/F228I				L	*	*						*	*						*	*
208	f	27	WNT10A:R113C	ar	22	16	U	*	*		*	*	*	*			*	*	*	*		*	*
			/E194AfsX28				u							*			*						
							l																
							L	*	*		*	*					*			*		*	*
242	f	16	WNT10A:R113C/-	ac	9	6	U	*				*	*	*			*	*		*			
			; EDARADD:S103F/-				L	*															*
299	f	54	WNT10A:R113C/-	ac	12	8	U	*			*	*							*	*			*
			; EDARADD:S103F/-				L	*			*	*							*	*			*
45	f	15	WNT10A:V145M/-	ar?	12	10	U				*			*			*			*			
							u							*			*						
							l																
							L				*			**c**	*	*	*		*	*			
210	f	22	WNT10A:V145L/-	ac	17	13	U	*			*	*		*			*		*	*			*
			; EDARADD:S103F/-				L	*	*		*			*			*		*	*		*	*
48	m	18	WNT10A:L154M/-	ad?	4	4	U													*			
							L							*	*		*						
42	m	11	WNT10A:G165R/-	?	34	18	U	*			*	*		*			*	*	*	*			*
							u					*	*	*				*	*	*			
							l					*	*		*	*		*	*				
							L	*			*	*	*	*	*	*	*	*	*	*		*	*
244	f	11	WNT10A:G165R/-	?	12	8	U	*			*	*							*	*			*
							L	*			*				*	*				*			*
259	f	37	WNT10A:G165R/-	?	9	6	U	*			*			*			*			*			
							L	*			*									*			*
277	m	11	WNT10A:G165R/-	ac	10	10	U	**?**			*	*		*			*		*	*			**?**
			; EDARADD:S103F/-				L	**?**			*	*							*	*			**?**
282	f	20	WNT10A:G165R/-	?	12	8	U	*			*	*		*			*		*	*			*
							L	*			*									*			*
207	m	11	WNT10A:	ar	15	11	U	*	*					*			**p**					*	*
			E222X/F228I				L	*	*				*	*	*	*	*	*				*	*
106	m	7	WNT10A:	ar	15	15	U	**?**			*		*	**p**			**p**	*	*	*			**?**
			F228I/F228I				L	**?**			*	*	*	*	*	*	*	*	*	*			**?**
206	f	33	WNT10A:	ar	15	11	U	*	*		*	*		*			*			*		*	*
			F228I/F228I				L	*	*		*									*		*	*
233	f	16	WNT10A:	ar	8	6	U				*	*							*	*			
			F228I/F228I				L	*			*									*			*
239	m	44	WNT10A:	ar	18	14	U	*	*		*	*		*			*			*		*	*
			F228I/F228I				L	*	*					*	*	*	*			*		*	*
243	m	43	WNT10A:	ar	10	7	U	*			*								*	*			
			F228I/F228I				L	*			*	*							*	*			*
270	f	12	WNT10A:	ar	22	18	U	*	*		*	*	*				*	*	*	*		*	*
			F228I/F228I				L	*	*		*	*			*	*		*	*	*		*	*
12	f	49	WNT10A:F228I/-	?	11	7	U	*			*	*								*			*
							L	*			*				*	*				*			*
51	m	9	WNT10A:F228I/-	ad?	4	4	U							*			*						
							L								*	*							
214	m	11	WNT10A:F228I/-	?	10	6	U	*			*	*							*	*			*
							L	*			*									*			*
217	m	10	WNT10A:F228I/-	?	15	11	U	*			*	*	*	*			*	*	*	*			*
							L	*			*								*	*			*
229	m	12	WNT10A:F228I/-	?	12	8	U	*			*	*							*	*			*
							L	*			*	*							*	*			*
246	f	9	WNT10A:F228I/-	ad?	6	6	U				*	*							*	*			
							L												*	*			
254	m	17	WNT10A:F228I/-	?	8	5	U	*						*			*						*
							L	*					*		*	*							
257	f	20	WNT10A:F228I/-	?	10	8	U	*			*	*					*		*	*			*
							L				*	*								*			
261	m	16	WNT10A:F228I/-	?	13	9	U	*					*	*			*	*					*
							L	*			*				*	*	*			*			*

* fam, family; f, female; m, male; n ta all, amount of missing teeth; n ta per, amount of missing permanent teeth excluding the third molars; ad, autosomal dominant; ar, autosomal recessive; Xl, X-linked; ac, combinations of alleles of different genes; U, maxillary permanent teeth; L, mandibular permanent teeth; u, maxillary deciduous teeth; l, mandibular deciduous teeth; p, peg-shaped; c, conical; s, abnormally small

**Figure 1:**
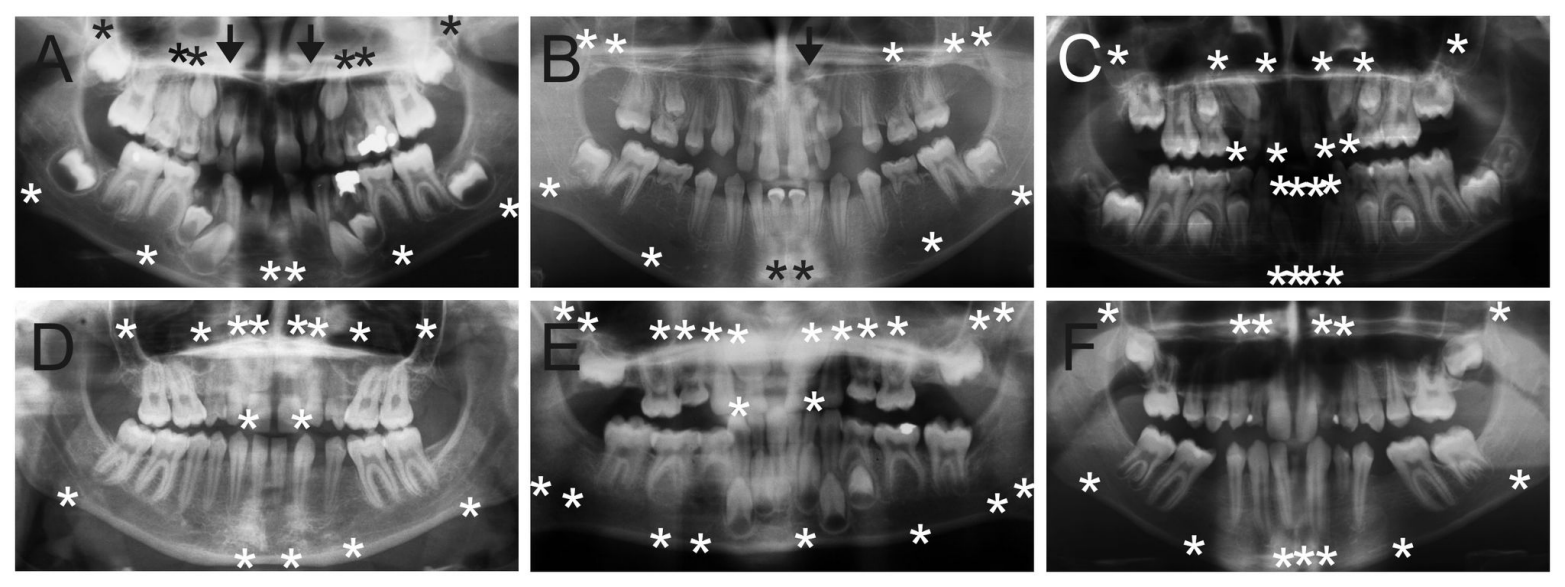
Panoramic radiographs of dentitions with tooth agenesis. Congenitally missing teeth are denoted with asterisks (*), and peg-shaped maxillary lateral incisors with arrows. All radiographs show many retained deciduous teeth. A. Dentition of a 15-year-old girl (family 219) with a heterozygous frameshift mutation K237SfsX2 in *MSX1*. All second premolars and third molars, maxillary first premolars and mandibular central incisors are missing. Maxillary lateral incisors are peg-shaped. **B**. 11-year-old girl (family 283) with a heterozygous missense mutation p. R47P in *PAX9* lacks all third molars, two maxillary second molars, three second premolars and mandibular central incisors. Left maxillary lateral incisor is peg-shaped. **C**. Severe tooth agenesis in a 13-year old boy (family 46) with a p.R357W mutation affecting the TNF domain of EDA. 10 permanent and 8 deciduous teeth, mostly in the anterior region, are missing. Maxillary left central incisor is conical. **D**. 19-year-old woman (family 266) lacks all third molars and lateral incisors, both maxillary canines, three second premolars and two deciduous lateral maxillary incisors. She was heterozygous for p.R325W in *EDAR*, p. S103F in *EDARADD* and p.G165R in *WNT10A*. **E**. Only 12 permanent teeth have developed in 27-year old woman (family 208) with a compound heterozygote mutations p.R113C and p.E194AfsX28 in *WNT10A*. Maxillary deciduous lateral incisors were also missing. **F**. 16-year old boy (family 261) with a heterozygous p.F228I variant in *WNT10A* lacks 13 permanent teeth including all third molars, mandibular second premolars, maxillary canines and five incisors.

Mutations in *MSX1* were detected in two families and in *PAX9* in four families, all in heterozygous state. In *MSX1*, two single nucleotide frameshift mutations, c.707delG (p. K237SfsX2) and a *de novo* c.665-666insA (p.N222KfsX118) were observed in exon 2 affecting the sequence downstream from the homeodomain. The patients lacked 12 or 14 permanent teeth, including all second premolars and most third molars (Figure 1A). All mutations in *PAX9*, two missense and two nonsense, resided in exon 2 and affected the paired domain (InterPro IPR001523). The probands lacked five and seven permanent teeth, mostly permanent molars, in addition to all third molars (Figure 1B). Bioinformatic analysis suggested that both missense mutations c.140G>C (p. R47P) and c.167C>T (p. I56T) disturb protein function. One of the nonsense mutations, c.340A>T (p.K114X), we have described previously in another Finnish family [27], but we were not able to detect relationship between the families.

We identified mutations in *EDA* in four probands. Three of them affected the TNF homology domain. A boy with c.1069C>T (p.R357W) had mild ectodermal symptoms like soft nails and atopic skin and lacked eight deciduous and eight permanent teeth, mostly incisors, in addition to two third molars (Figure 1C). The mutation originated in his unaffected mother. In a girl who lacked six permanent incisors and two first premolars, we detected a heterozygous mutation c.1133C>T (p.T378M). Bioinformatic analysis suggested that both variants have a significant effect on protein function. A heterozygous truncating frameshift mutation c.901-904delTACT (p.T301SfsX5) was observed in a 6-year-old girl who had a very severe tooth agenesis phenotype affecting both permanent and deciduous dentition. Finally, a boy lacking three permanent and two deciduous incisors had a *de novo* deletion of 18 base pairs in the collagen-like domain (c.612-629del, p.IPGIPG205-210del).

Four different missense mutations in altogether eight families, all in heterozygous state, were observed in exons 11 and 12 of *EDAR*. These mutations affect the intracellular part of the protein and bioinformatic tools suggested variable extent of harmful effects on protein function. c.973C>T (p.R325W) and c.1135G>A (p.E279K) were both detected in three families, whereas c. 1073G>A (p.R358Q) and c.1172T>A (p.M391K) were present in single families. p.M391K was detected in a father and daughter who both lacked eight permanent teeth. Two other probands lacked more than five permanent teeth while in five other probands tooth agenesis was essentially limited to the incisor region with two to five permanent incisors missing. We could verify agenesis of at least one deciduous incisor in seven of the probands. One of the probands with a severe phenotype (family 266) carried heterozygous alterations in *EDARADD* (p. S103F) and *WNT10A* (p.G165R) in addition to p.R325W in *EDAR* (Figure 1D). Combination of the same *EDAR* and *EDARADD* variants was also observed in the proband of family 52, and the *EDAR* p.R325W was inherited from an affected grandmother and unaffected mother. In two other families (47 and 67), both affected and unaffected heterozygous relatives were observed. In family 49, both parents had a mild phenotype, but the identified mutation was inherited from the father.

Our work also revealed that altogether 17 unrelated persons, 10 of the probands and 7 unaffected controls, carried the c.1109C (p. 370 alanine) allele in heterozygous state. The allele frequency of this allele as calculated from unrelated samples would be 3.3% which suggests higher frequency of this allele than has been reported for European populations. This allele has been reported to originate in China some 35000 years ago and be associated with increased hair thickness and shovel-shaped incisors [29,30]. None of our heterozygotes had this dental feature.

In *EDARADD* we detected two variants in exon 7 that caused an amino acid change and both were predicted to be harmful for protein function by bioinformatic analysis. c.508C>T (p.R170W) was present in a heterozygous state in a proband of family 209 who lacked all third molars and seven other permanent teeth. c. 308C>T (p. S103F) was present in altogether 11 probands (Table 2), but also in four of our unaffected control samples. Phenotypes in nine probands fulfilled the criteria of oligodontia. Seven of the probands also had heterozygous mutations in either *EDAR* or *WNT10A*.

In *WNT10A* we detected altogether nine different variants, three of which have to our knowledge not been described earlier. No remarkable signs in other ectodermal organs were recorded in these patients. Nonsense mutation c.664G>T (p.E222X) was present together with a missense variant c.682T>A (p.F228I) in two affected siblings and a frameshift-causing 14 bp deletion at c.579-592 (p.E194AfsX2) with a missense variant c.337C>T (p.R113C) in another proband (Figure 1E). Six probands were homozygous for p.F228I, one proband homozygous for p.R113C and two probands compound heterozygous for these variants. All 11 probands with biallelic genotypes had severe tooth agenesis with mean number of missing teeth of 14.4. The phenotypes showed a significant variation: range of missing teeth was from 8 to 22 and no distinct pattern of missing teeth was observed. We could detect agenesis of any deciduous teeth only in one of these probands.

p. R113C was also detected in heterozygous state in one and p.F228I in five probands (Figure 1F). c.208C>T (p. R70W) was detected in heterozygous state in two affected brothers of family 276 lacking 10 and 5 permanent teeth. The brother with the more severe phenotype carried also the EDARADD p. S103F variant. c.443G>A (p.V145M) was detected in heterozygous state in seven members of a single family (45), two of whom were affected with a rather severe phenotype (12 and 11 missing teeth) and one lacking four permanent teeth. A novel variant affecting the same amino acid, c.443G>T (p.V145L) was found in a proband with 13 missing teeth. c.460C>A (p.L154M) was detected in a proband with agenesis of four permanent teeth as well as in four of his family members two of whom were unaffected. All of the missense variants mentioned above were predicted to be harmful for the protein function. Altogether we detected heterozygous genotypes of these variants of *WNT10A* in 15 probands, 13 of whom classified as oligodontia. Mean number of missing teeth for these heterozygous probands was 10.4 (range 4 to 17). Heterozygous genotypes were also observed in 30 relatives of all probands, 12 of whom were unaffected and 18 affected, with a mean of 2.4 missing teeth (range 0 to 11).

In addition, a previously described variant, c.493G>A (p.G165R), was detected in heterozygous state in six probands with oligodontia. Bioinformatic analysis by SIFT and PolyPhen2 suggests that this variant is benign, which was also suggested by earlier observations [17]. However, we did not detect this variant among our controls. Furthermore, the amino acid change destroys an RGD motif, a binding site for integrins [31]. The change also creates a stretch of three consecutive arginines, which has been recognized as a cytoplasmic signal for retaining proteins in the endoplasmic reticulum [32].

Because of the large amount of heterozygous genotypes of *WNT10A* was observed among the probands with relatively severe tooth agenesis phenotype, in most cases comparable to the phenotypes associated with biallelic genotypes, we reasoned that these patients may harbor another mutation that could not be revealed by direct sequencing of the exons. Testing this hypothesis, we did not detect any alterations in the immediate promoter (920 bp upstream of the start codon) of *WNT10A* or in the paralogous genes *WNT10B* and *WNT6*, the orthologues of which are co-expressed with *Wnt10a* in the early mouse dental epithelium [24]. We also performed long range PCR over exons 1 and 2 and over exons 3 and 4 followed by nested sequencing and could in most cases detect heterozygosity, either for the exonic mutations or for intronic polymorphisms (rs74333950 IVS1+462T>G, rs10177996 IVS1-322T>C, and rs7349332 IVS3-1113), thus excluding possibility of whole exon deletions (data not shown). However, several of these probands carried also a heterozygous *EDARADD* p. S103F variant (Table 3).

## Discussion

In this report, we have described mutational analysis of seven candidate genes for tooth agenesis in the Finnish patient cohort. This cohort has been recruited during a long time period among the patients visiting the specialist dental clinics of the University of Helsinki or Helsinki University Central Hospital. Especially the probands with less severe phenotypes are not a representative sample but they were included in this report because of the mutations we observed in some of them and because we felt it arbitrary to set an inclusion criteria to a certain number of missing teeth.

### Nature of the identified mutations

Frameshift and nonsense mutations are highly likely all causative because they involve profound alteration of the protein primary structure. The two mutations in *MSX1* are the first mutations that affect the C-terminal part of the protein downstream of the homeodomain the function of which is poorly understood. The in-frame deletion in the collagen-like domain observed in *EDA* is similar albeit not identical to several previously reported mutations in HED families [33–35].

We excluded missense variants that were present with high frequencies in our patient and unaffected control cohorts or in the dbSNP or 1000genomes databases. The remaining missense variants were almost all suggested to be harmful by the bioinformatic tools and most of them affected known functional domains. The two missense changes in *PAX9* affected the highly conserved paired domain and are expected to affect the DNA-binding properties of this domain. The EDA p.R357W and p.T378M affect the TNF homology domain (InterPro IPR006052) of this extracellular signaling protein, and similar mutations in this domain have previously been shown to cause tooth agenesis in males who carry a single copy of this X-chromosomal gene and in part of heterozygous female carriers [14,16,36]. The *EDAR* missense mutations reside in the intracellular death and death-like domains (InterPro IPR000488 and IPR011029) important for receptor multimerization and the interaction with the adapter protein EDARADD essential for signal transduction. The EDARADD p.R170W resides in the death domain interacting with EDAR. Previously, both homozygous and heterozygous mutations affecting these domains have been described in families with obvious recessive and dominant inheritance of HED, respectively (OMIM 129490 and 614941) [34,37–40]. We suggest that p.M391K which was detected in a father and daughter with a severe tooth agenesis and perhaps also the EDARADD p.R170W have some extent of dominant negative effect. The other mutations in *EDAR* were associated with more variable and usually less severe phenotype and some relatives were unaffected. We suggest that these amino acid changes essentially cause loss of function that causes tooth agenesis with incomplete penetrance and that the patients express carrier phenotypes of autosomal recessive HED (OMIM 224900). Indeed, the p.E379K mutation is homologous to the original mutation described in d*ownless* (*dl*), the mouse model of autosomal recessive HED [41]. EDARADD p. S103F was reported in heterozygous state earlier in a single patient who lacked six permanent teeth [42]. We detected this variant in heterozygous state in 11 probands but also in some unaffected controls, suggesting low penetrance if present isolated in heterozygous state.

Based on alignment with XWnt8, the *Xenopus* Wnt protein whose three-dimensional structure and interactions with the Frizzled receptor has been elucidated by X-ray crystallography [43], all missense variants of *WNT10A* affect the N-terminal domain which consists of an alpha-helical bundle. p.F228I homozygous and heterozygous genotypes were observed in this and earlier studies on ectodermal dysplasia syndrome and isolated tooth agenesis [17,18,22,38] in striking contrast with the prevalences among the controls. p.R113C was detected in several probands but not in controls and it was previously found in a compound heterozygote patient [19]. We observed p. R70W, p. V145M, p.V145L and p.L154M only in single families and only in heterozygous state but they were also predicted as harmful by both SIFT and PolyPhen2. p.V145M has been described earlier in patients [18,38]. A previously known variant p.G165R was predicted as benign, and was previously observed in trans together with p.F228I in unaffected persons [17]. However, the presence in six patients with severe tooth agenesis in our cohort but not in controls, and the consequences of the amino acid change on the level of the protein primary structure, suggest that association of this variant with abnormal tooth development should not be excluded.

Several of the probands who had heterozygous genotypes of *WNT10A* had equally severe phenotypes as those with biallelic genotypes. Heterozygous genotypes of *WNT10A* were described also in earlier reports in association of syndromic phenotypes or isolated severe tooth agenesis [18,19,22,38]. Phenotypic variation is inherent in tooth agenesis but we consider it plausible that the extreme variation observed in these cases reflects existence of another mutation. In two of our families all affected relatives did not have the mutation detected in the proband, and these included sons of the proband in family 12 and mother of the proband in family 261 (Table 2). In these and several other families we could not identify any alterations in the promoter and excluded whole exon deletions in *WNT10A* as well as coding region mutations in the co-expressed *WNT10B* and *WNT6*. However, we observed the *EDARADD* mutation, p. S103F, in several probands with heterozygous *WNT10A* genotypes, as well as in two with heterozygous genotypes of *EDAR*. Most of the probands with these allele combinations had a severe phenotype as compared to the other probands or relatives who had only the *EDAR* or *WNT10A* allele. One of the probands (family 266) was actually heterozygous for three variants, EDAR p.R325W, EDARADD p. S103F and WNT10A p.G165R, consistent with the most severe tooth agenesis observed among the *EDAR* heterozygotes. Combinations of two mutations affecting the EDA pathway, i.e. the EDA receptor and the adapter protein EDARADD, may be expected to have a stronger effect than a single mutation even though the combination shall not affect the pathway to a similar extent as biallelic mutations in one gene. It would neither be surprising if combinations of harmful albeit heterozygous mutations in EDA and WNT pathways would cause additive effects. It is known that both pathways have an instrumental role in the dental epithelium from the early stages of tooth development and it is known that they interact during dental and hair development [44,45]. This is the first molecular observation of combined effects of mutations in distinct genes on tooth agenesis, implying that monogenic inheritance does not explain all cases of tooth agenesis.

### Contributions to tooth agenesis

Overall, we have described mutations in 58 probands of our Finnish patient cohort, including our previous reports on two families with mutations in *PAX9* and two with mutations in *AXIN2* [13,26,27]. These account for 44% of all our probands. However, in one third of the probands the observed genotype of *WNT10A* or *EDARADD* may not alone explain the phenotype. The significant majority of these probands had a severe phenotype, and most of them fulfilled the criterium for oligodontia (agenesis of six or more permanent teeth, excluding third molars). Several of the other probands had a specific phenotype that involved both permanent and deciduous incisors. Only a few of the probands with identified mutations conformed to the typical incisor premolar hypodontia [25] and no mutations were detected in the probands or other members of the multiplex families in which the latter phenotype is inherited in an autosomal dominant manner [25]. However, this kind of phenotypes were observed in several heterozygote relatives of the probands with oligodontia.

Considering our whole patient cohort, i.e. including the previous reports, we have detected mutations in *PAX9* in six probands, and two in both *AXIN2* and *MSX1*, all with severe phenotypes. Our results are in line with reports from the earlier reports in which population-based patient cohorts of severe tooth agenesis have been analysed for candidate gene mutations [18,42], supporting the conclusion that dominant mutations in these genes explain severe tooth agenesis in a relatively minor proportion of all families. However, the relative contribution of these three genes with dominant mutations to severe tooth agenesis becomes more significant if all patients, and not only probands, are considered. Thus, in our whole patient cohort, severe tooth agenesis is explained in altogether 13 patients by mutations in *PAX9* and in 11 by mutations in *AXIN2*.

We detected mutations in *EDA* in four probands, three with severe phenotypes. While *EDA* mutations are revealed as a cause of isolated tooth agenesis, in female carriers or as hypomorphic mutations in males often appearing recessive or sporadic, they appear to have a minor contribution [18,42]. However, our results suggests that the whole contribution of EDA pathway is more significant, considering also the mutations in the *EDAR* and *EDARADD* genes. To our knowledge, this is the first report describing mutations in *EDAR* in isolated tooth agenesis. They were detected in eight probands, three of whom had a severe phenotype. Heterozygous EDARADD p. S103F variant was detected in 11 of our probands, most with a severe phenotype. Altogether this variant was present in 15 unrelated samples, 11 affected and four unaffected, which is in line with the reported allele frequency of 2% in the dbSNP database. Thus, this variant has potentially a significant contribution to tooth agenesis on the population level.

We found biallelic genotypes of *WNT10A* in 11 probands, corresponding to 8.4% of all probands, and 13% of those with a phenotype defined as oligodontia. Heterozygote genotypes of *WNT10A* – including p.G165R - were observed in 19 probands with severe tooth agenesis and two other probands, which increased involvement of this gene to 23% of all probands and to 35% of those with a severe phenotype. We also detected several affected, most with typical incisor premolar hypodontia phenotype, as well as unaffected heterozygote relatives, consistent with reduced penetrance of these alleles. Thus, *WNT10A* has the most significant contribution of so far identified genes to tooth agenesis, in line with previous reports [18]. Several of the mutations have been detected in multiple studies and thus the high contribution of *WNT10A* to tooth agenesis is most apparently based on the presence, and even with moderate frequencies, of the disease causing alleles in all studied populations. However, the contribution reported here is remarkably smaller than in recent reports from the Netherlands and Poland [18,19]. Our cohort was larger than in the previous studies and includes more probands with less severe phenotypes (mean number of missing teeth of 9.3 in oligodontia as compared to 14.6 in the Dutch cohort). In addition, it appears that there are differences in the allele frequencies between different studied populations. We have detected no cases of a common nonsense allele, p.C107X, in our cohort or controls. The Dutch study reported a frequency of 2.3% and the Polish study a frequency of 1,3% for the most common disease allele, p.F228I [18,19]. dbSNP database reports similar frequencies for this allele in two large cohorts but the German study reported a significantly lower frequency of 0.5% [17]. We did not detect this allele once among the more than 100 unaffected controls, suggesting frequency similar to the German study in the Finnish population.

What are the implications of our results for the genetic background of the common types of tooth agenesis, especially the common hypodontia, which involves agenesis of one or a few permanent incisors and second premolars and most often follows dominant inheritance [25,46]? We or others have not identified coding region mutations in *MSX1*, *PAX9* and *AXIN2* associated with this common phenotype. However, putative associations of several silent or non-coding region variants to common hypodontia have been reported [47,48]. The results from this and earlier studies show that several variants of *EDAR* and *WNT10A* in heterozygous state contribute to incisor premolar hypodontia phenotypes with a penetrance that conforms to dominant inheritance. This kind of alleles – and also in other genes – connect the genetic background of common hypodontia and severe tooth agenesis: the alleles that in heterozygous state cause common hypodontia by dominant inheritance, cause severe tooth agenesis when present as biallelic recessive genotypes or allele combinations of different genes. However, they were not present in most of our probands with a hypodontia phenotype and especially not in the families in which we have described dominant inheritance [25], suggesting that they have a minor contribution to these phenotypes.

### Gene specific effects on phenotypes

Mammalian teeth are divided into different classes according to their morphology and position on the dental arches. Despite increasing knowledge of the genetic mechanisms underlying tooth development, and even of the regulatory mechanisms important in modulating the cusp pattern and complex morphologies of multicusped teeth [49], the genetic basis providing different morphologies (heterodonty) in mammalian dentitions has not been elucidated.

As supported by our results, heterozygous loss of function mutations in *MSX1* and *PAX9* have a strongest effect on the posterior multicusped teeth. *MSX1* mutations affect almost uniformly all second premolars and third molars, while permanent molars are the most sensitive to *PAX9* mutations [8,12]. In HED, even complete anodontia may sometimes be observed as a consequence of complete inactivation of the EDA signaling pathway, but in female carriers of the X-linked mutations, the tooth agenesis is most prominent in the anterior dentition [50]. The hypomorphic *EDA* mutations have an even more specific effect on both deciduous and permanent incisors in both sexes [14,16]. The phenotypes of our male and female patients with mutations in *EDA* conform to this tendency but it is also recapitulated by the phenotypes associated with heterozygous mutations in *EDAR*. However, this feature was not observed in the patients with heterozygous mutations in *EDARADD*.

Compilation of the data on tooth specific phenotypes associated with biallelic *WNT10A* mutations [17–19,22] shows that these mutations affect all tooth types, in line with the expression of the mouse homolog in both incisor and molar tooth germs [23]. Most commonly affected are the third molars, and after them second premolars and maxillary lateral and mandibular incisors whereas the first permanent molars and maxillary central incisors are most stable. This order follows the prevalences of agenesis of different tooth types in general [4]. However, frequencies of missing canines, first premolars and second permanent molars are also quite high among the patients with biallelic *WNT10A* mutations. Furthermore, there is significant variation between patients in the severity of agenesis and in the tooth types that are affected. As a further distinction to EDA pathway, deciduous teeth are usually not affected in the non-syndromic patients who most often carry missense than stop-codon creating mutations that are more common among syndromic patients [18,21,38,51].

The differential sensitivities of specific parts of dentition for reduced doses of genes may be dependent on differential expression levels of these genes and they may reflect different roles of the genes during normal development. Posterior, multicusped teeth may be especially sensitive to the role of *MSX1* and *PAX9* as regulators of the recruitment of the mesenchyme to dental fate and the regulation of mesenchymal signaling necessary to the cascade of the morphogenetic epithelial signaling centers enamel knots [49,52]. On the other hand, EDA signaling regulates the size of the ectodermal signaling centers [53,54], which may be critical for the development of anterior dentition. *Wnt10a* expression during mouse dental development is largely superimposed to that of *Edar* [23], but its target cells are not known. Despite the regulatory interaction between these pathways, the differences in human phenotypes suggests that *WNT10A* deficiency exerts its effect through different mechanisms than EDA signaling.

## Conclusions

In this work, we have performed mutational analysis of seven candidate genes in our cohort of tooth agenesis. Mutations were identified 44% of the probands, mostly in association with severe phenotypes. However, the phenotypes of several of the heterozygous relatives show that some of the mutations also explain a minor fraction of common incisor premolar hypodontia. The relative contribution of *WNT10A* was highest but less prominent than in recent reports from smaller cohorts. Together, mutations affecting genes of the EDA signaling pathway was also remarkable. Our results suggest that some of the phenotypes are explained by combined effects of alleles in distinct genes.

## Supporting Information

Table S1 Primer sequences and PCR and sequencing reaction conditions. (XLSX)

Table S2 Candidate gene genotypes and tooth agenesis phenotypes of relatives. (XLSX)
